# A Coral-Derived Compound Improves Functional Recovery after Spinal Cord Injury through Its Antiapoptotic and Anti-Inflammatory Effects

**DOI:** 10.3390/md14090160

**Published:** 2016-09-02

**Authors:** Chun-Hong Chen, Nan-Fu Chen, Chien-Wei Feng, Shu-Yu Cheng, Han-Chun Hung, Kuan-Hao Tsui, Chi-Hsin Hsu, Ping-Jyun Sung, Wu-Fu Chen, Zhi-Hong Wen

**Affiliations:** 1 Doctoral Degree Program in Marine Biotechnology, National Sun Yat-Sen University, Kaohsiung 80424, Taiwan; anubis0620@gmail.com (C.-H.C.); qscjuejuejue@gmail.com (C.-W.F.); joygetit@gmail.com (S.-Y.C.); hanchun25@gmail.com (H.-C.H.); hsuch@mail.nsysu.edu.tw (C.-H.H.); 2 Doctoral Degree Program in Marine Biotechnology, Academia Sinica, Taipei 11529, Taiwan; 3 Division of Neurosurgery, Department of Surgery, Kaohsiung Armed Forces General Hospital, Kaohsiung 80284, Taiwan; chen06688@gmail.com; 4 Department of Neurological Surgery, Tri-Service General Hospital, National Defense Medical Center, Taipei 11490, Taiwan; 5 Department of Obstetrics and Gynecology, Kaohsiung Veterans General Hospital, Kaohsiung 81362, Taiwan; khtsui60@gmail.com; 6 Department of Obstetrics and Gynecology and Institute of Clinical Medicine, National Yang-Ming University, Taipei 11221, Taiwan; 7 Department of Pharmacy and Graduate Institute of Pharmaceutical Technology, Tajen University, Pingtung County 90741, Taiwan; 8 Department of Marine Biotechnology and Resources, National Sun Yat-Sen University, Kaohsiung 80424, Taiwan; pjsung@nmmba.gov.tw; 9 National Museum of Marine Biology & Aquarium, Pingtung 94450, Taiwan; 10 Graduate Institute of Marine Biology, National Dong Hwa University, Pingtung 94450, Taiwan; 11 Department of Neurosurgery, Kaohsiung Chang Gung Memorial Hospital and Chang Gung University College of Medicine, Kaohsiung 83301, Taiwan

**Keywords:** *Sinularia flexibilis*, marine natural compound, 11-dehydrosinulariolide, microglia, neuroprotection, spinal cord injury, anti-inflammation

## Abstract

Background: Our previous in vitro results demonstrated that 11-dehydrosinulariolide significantly reduced 6-hydroxydopamine-induced cytotoxicity and apoptosis in a human neuroblastoma cell line, SH-SY5Y, and suppressed the expression of inducible NO synthase (iNOS) and cyclooxygenase 2 in lipopolysaccharide-stimulated macrophage cells. The neuroprotective and anti-inflammatory effects of 11-dehydrosinulariolide may be suitable for treating spinal cord injury (SCI). Methods: In the present study, Wistar rats were pretreated with 11-dehydrosinulariolide or saline through intrathecal injection after a thoracic spinal cord contusion injury induced using a New York University (NYU) impactor. The apoptotic cells were assessed using the terminal deoxynucleotidyl transferase dUTP nick end labeling (TUNEL) assay. The expression and localization of proinflammatory, apoptosis-associated and cell survival-related pathway proteins were examined through immunoblotting and immunohistochemistry. Results: 11-Dehydrosinulariolide attenuated SCI-induced cell apoptosis by upregulating the antiapoptotic protein Bcl-2 and cell survival-related pathway proteins p-Akt and p-ERK, 8 h after SCI. Furthermore, the transcription factor p-CREB, which regulates Bcl-2 expression, was upregulated after 11-dehydrosinulariolide treatment. On day 7 after SCI, 11-dehydrosinulariolide exhibited an anti-inflammatory effect, attenuating SCI-induced upregulation of the inflammatory proteins iNOS and tumor necrosis factor-α. 11-Dehydrosinulariolide also induced an increase in the expression of arginase-1 and CD206, markers of M2 microglia, in the injured spinal cord on day 7 after SCI. Thus, the anti-inflammatory effect of 11-dehydrosinulariolide may be related to the promotion of an alternative pathway of microglia activation. Conclusion: The results show that 11-dehydrosinulariolide exerts antiapoptotic effects at 8 h after SCI and anti-inflammatory effects at 7 days after SCI. We consider that this compound may be a promising therapeutic agent for SCI.

## 1. Introduction

During central nervous system (CNS) insults, microglia are the first responders; they produce proinflammatory mediators such as chemokines and nitric oxide (NO) [1]. These mediators contribute to eliminating pathogens as well as activating astrocytes and neurons, which subsequently activate microglia [2]. Previous studies have reported that the implantation of activated microglia or macrophages into an injured rat spinal cord could promote axon regeneration after spinal cord injury (SCI) [3,4]. This acute inflammation is beneficial to the CNS for limiting the injury region, performing neurogenesis, and repairing damaged tissues [5,6]. These results suggest that microglial activation may exert beneficial effects after SCI. However, long-term activation of microglia and astrocytes results in pathological forms of inflammation, which have critical roles in several neurodegenerative diseases [7].

To limit the inflammatory region, astrocytes become reactive and form a dense cell plexus. They also express an extracellular matrix, which forms a glial scar after SCI [8,9]. However, the physical and chemical characteristics of the glial scar limit neurite outgrowth and axonal regeneration after SCI [10,11]. Therefore, limiting the inflammatory area after SCI reduces neuronal death and attenuates glial scar formation, thus providing a therapeutic effect.

The CNS contains two microglia subtypes: the classically activated M1 phenotype and the alternatively activated M2 phenotype [12,13,14]. M1 microglia release proinflammatory factors [15,16], whereas M2 microglia exert a neuroprotective effect by expressing high levels of interleukin (IL)-10 and tumor growth factor-β (TGF-β) and downregulating proinflammatory cytokine expression [16,17,18]. After SCI, M1 microglia contribute to increasing the production of oxidative metabolites and proinflammatory cytokines for host defense. In contrast, M2 microglia promote wound healing and suppress destructive immune responses [19]. After SCI, the number of M2 microglia is relatively lower than that of M1 microglia, and this lower number may contribute to prolonged proinflammatory responses [12]. Therefore, several studies have reported that increasing M2 microglia polarization enhances recovery after SCI [13,14,20,21].

11-Dehydrosinulariolide is a cembrane-type compound, and most cembrane-type compounds were isolated from marine organisms, particularly the soft coral *Sinularia*. 11-Dehydrosinulariolide was first isolated from *Sinularia flexibilis* [22]. The 11-dehydrosinulariolide used in the present study was isolated from cultured *S. flexibilis* at Taiwan’s National Museum of Marine Biology and Aquarium. Our previous in vitro results demonstrated that 11-dehydrosinulariolide significantly reduced 6-hydroxydopamine-induced cytotoxicity and apoptosis in a human neuroblastoma cell line, SH-SY5Y, and suppressed the expression of proinflammatory proteins, inducible NO synthase (iNOS) and cyclooxygenase-2 (COX-2), in lipopolysaccharide-stimulated macrophage cells [23]. Furthermore, 11-dehydrosinulariolide-enhanced p-Akt upregulation in 6-hydroxydopamine-treated SH-SY5Y cells may be associated with antiapoptotic activity. Thus, because of its neuroprotective and anti-inflammatory effects, 11-dehydrosinulariolide may be suitable for SCI treatment. In the present study, we examined the neuroprotective and anti-inflammatory effects of 11-dehydrosinulariolide in a rat SCI model.

## 2. Results

### 2.1. 11-Dehydrosinulariolide Improved Locomotor Behavior after SCI

**Figure 1:**
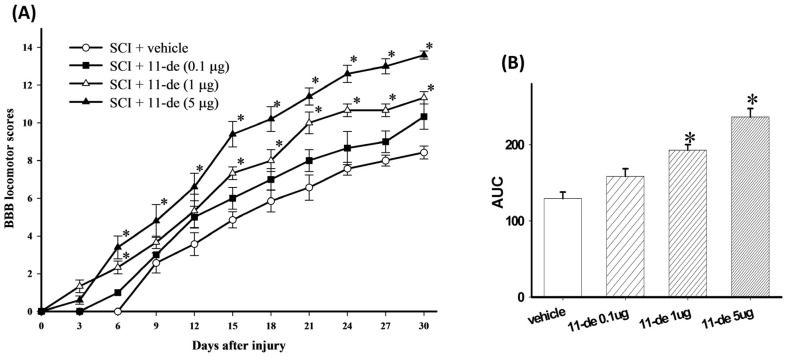
I.t. administration of 11-dehydrosinulariolide improved locomotor recovery in rats with SCI. After SCI, locomotor behavior was evaluated using the BBB score every 3 days. Significant improvement was observed in hindlimb motor function with 1 and 5 μg 11-dehydrosinulariolide-treated rats after SCI (**A**). The area under the BBB score-time curve for 1 and 5 μg 11-dehydrosinulariolide-treated rats indicated significant improvements compared with those administered the vehicle (**B**). Data are expressed as means ± SEMs; * *p* < 0.05 compared with the vehicle group.

11-Dehydrosinulariolide was administered to the SCI rats through intrathecal (i.t.) injection (pretreatment for 2 days and once a day after SCI for 5 days) at 3 doses (0.1, 1, or 5 μg/10 μL). The 0.1-μg group had more satisfactory Basso-Beattie-Bresnahan (BBB) scores compared with the vehicle group after SCI, but the difference was not statistically significant (Figure 1). Nevertheless, 1 μg of 11-dehydrosinulariolide significantly inhibited SCI-induced neurological dysfunction from day 15 to day 30 after SCI. The rats in the 5-μg group showed significant improvements in the BBB scores compared with the vehicle-treated rats from day 6 to day 30 after SCI. After the transformation of behavioral data to areas under BBB score-time curves (AUCs), rats receiving 1- and 5-μg 11-dehydrosinulariolide treatments showed significant alleviation of SCI-induced motor dysfunction compared with those receiving the vehicle (Figure 1B). These results demonstrate that 11-dehydrosinulariolide significantly alleviated SCI-related dysfunctions. Moreover, the AUC of the 5-μg group was significantly higher than that of the 1-μg group. Therefore, we employed the 5-μg dose for subsequent experiments. In our preliminary test we administered 11-dehydrosinulariolide immediately after SCI and on the following 6 days (once daily; total, 7 times; *n* = 2). Co-treatment with 11-dehydrosinulariolide showed therapeutic effects after SCI (Supplementary Materials Figure S1A); however, the AUC data showed that the co-treatment was not as effective as the pretreatment at the same dose (Supplementary Materials Figure S1B).

### 2.2. Antiapoptotic Effects of 11-Dehydrosinulariolide after SCI

**Figure 2:**
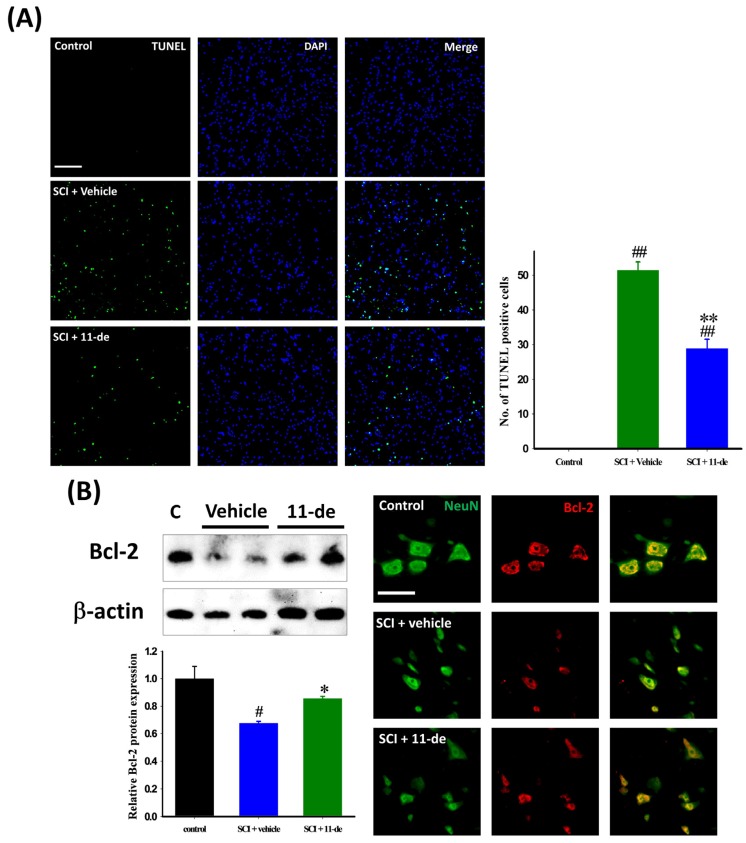
11-Dehydrosinulariolide reduced apoptosis after SCI. The rats were sacrificed 8 h after SCI, and their spinal cords were harvested. The apoptotic cells were identified through a TUNEL assay (**A**); Cells with the TUNEL signal (**green**), which colocalized with DAPI (**blue**), were counted as apoptotic. 11-Dehydrosinulariolide significantly reduced the number of apoptotic cells after SCI. 11-Dehydrosinulariolide administration attenuated SCI-induced Bcl-2 downregulation (**B**); The neuronal Bcl-2 IR signal in the 11-dehydrosinulariolide group was stronger than that in the SCI group. Data are expressed as means ± SEMs; # *p* < 0.05 and ## *p* < 0.001 compared with the control group; * *p* < 0.05 and ** *p* < 0.001 compared with the vehicle group. Scale bars = 100 μm in (**A**), 50 μm in (**B**).

To examine the antiapoptotic effects of 11-dehydrosinulariolide, the rats were sacrificed 8 h after SCI, a time point used in previous studies [24,25]. The terminal deoxynucleotidyl transferase dUTP nick end labeling (TUNEL) assay and 4′,6-diamidino-2-phenylindole (DAPI) staining were performed on spinal cord sections for identifying apoptotic cells after SCI. Images were taken at the lesion epicenter (Figure 2A). The number of TUNEL-positive cells in the vehicle group was 51.42 ± 2.43; but only 28.83 ± 2.72 in the group treated with 11-dehydrosinulariolide. Next, we examined the expression of the antiapoptotic protein Bcl-2 after SCI. Compared with the control group, Bcl-2 expression was downregulated after the SCI (Figure 2B). 11-Dehydrosinulariolide administration (i.t.) attenuated the SCI-induced downregulation of Bcl-2 expression. In the control spinal cord, the Bcl-2 immunoreactive (IR) signal was colocalized with the IR signal of the neuron-specific marker NeuN. Our immunohistochemistry images corroborate immunoblot findings. 11-Dehydrosinulariolide attenuated the SCI-induced downregulation of the neuronal Bcl-2 IR signal.

### 2.3. Effects of 11-Dehydrosinulariolide on the Neuroprotection-Related Signaling Pathway

**Figure 3:**
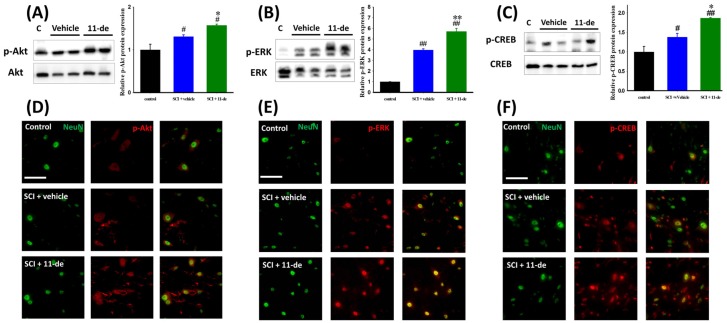
11-Dehydrosinulariolide enhanced the SCI-induced activation of cell survival-related signaling proteins, Akt and ERK. The rats were sacrificed 8 h after SCI, and their spinal cords were harvested to perform immunoblotting (**A**–**C**) and immunohistochemistry (**D**–**F**). 11-Dehydrosinulariolide (i.t.) significantly enhanced the SCI-induced activation of neuronal Akt, ERK, and CREB. Data are expressed as means ± SEMs; # *p* < 0.05 and ## *p* < 0.001 compared with the control group; * *p* < 0.05 and ** *p* < 0.001 compared with the vehicle group. Scale bars = 50 μm.

In a previous study, we reported that the in vitro neuroprotection effect of 11-dehydrosinulariolide is mediated through the regulation of phosphatidylinositol 3-kinase (PI3K) [23]. To further investigate the pathways involved in this neuroprotection effect after SCI, we performed immunoblotting and immunohistochemistry. The rats were sacrificed 8 h after SCI. We observed that 11-dehydrosinulariolide enhanced the SCI-induced phosphorylation of the cell survival-related pathway proteins Akt and ERK (Figure 3A,B,D,E). Immunoblotting results indicated that 11-dehydrosinulariolide enhances SCI-induced Akt and ERK phosphorylation (Figure 3A,B). According to the immunohistochemistry of the control spinal cord, the p-Akt IR signal was colocalized with the NeuN IR signal. After SCI, the neuronal p-Akt IR signal increased and some nonneuronal p-Akt IR signals were observed (Figure 3D). After administration of 11-dehydrosinulariolide, both neuronal and nonneuronal p-Akt IR signals were enhanced (Figure 3D). The p-ERK IR signal in the control spinal cord was weak and difficult to visualize; after SCI, this IR signal was upregulated and majorly colocalized with the NeuN IR signal. 11-Dehydrosinulariolide also enhanced the SCI-induced upregulation of the p-ERK IR signal. The transcription factor cyclic-AMP response element-binding protein (CREB) is a positive regulator of Bcl-2 induction [26,27,28,29]; therefore, we examined the level of phosphorylated CREB (p-CREB) after SCI (Figure 3C,F). 11-Dehydrosinulariolide significantly enhanced the SCI-induced upregulation of p-CREB (Figure 3C). In the control spinal cord, the p-CREB IR signal was majorly colocalized with neuronal signals. After SCI, the p-CREB IR signal was enhanced and some nonneuronal p-CREB IR signals were observed (Figure 3F). Thus, 11-dehydrosinulariolide enhances both neuronal and nonneuronal p-CREB IR signals.

### 2.4. 11-Dehydrosinulariolide Increased the Amount of White Matter Spared after SCI

**Figure 4:**
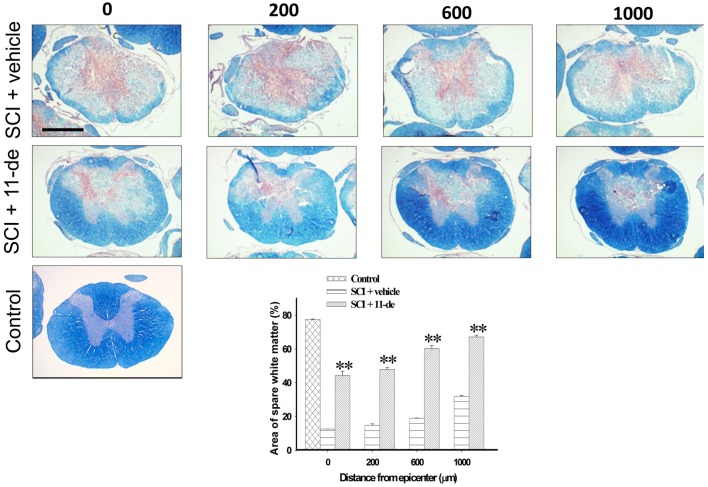
11-Dehydrosinulariolide administration increased the amount of white matter spared after SCI. The rats were sacrificed on day 7 after SCI. Spinal cord sections were stained with myelin-specific eriochrome cyanine-R (**blue**) and counterstained with neutral red (**red**). 11-Dehydrosinulariolide administration (i.t.) significantly enhanced the spare white matter area 200, 600, and 1000 μm rostral from the lesion epicenter. Data are expressed as means ± SEMs; ** *p* < 0.001 compared with the vehicle group. Scale bars = 1 mm.

To estimate the amount of white matter spared after SCI, spinal cord sections were stained with myelin-specific eriochrome cyanine R (blue) and counterstained with neutral red (red). The rats were sacrificed on day 7 after SCI, their spinal cords were sectioned continuously, and the section with the largest lesion was defined as the lesion epicenter. Figure 4 shows that the spinal cord sections with spared white matter are 200, 600, and 1000 μm rostral to the lesion epicenter. 11-Dehydrosinulariolide administration significantly enhanced tissue sparing on day 7 after SCI. Therefore, 11-dehydrosinulariolide treatment reduces demyelination in an injured spinal cord.

### 2.5. 11-Dehydrosinulariolide Attenuated SCI-Induced Inflammatory Protein Expression

**Figure 5:**
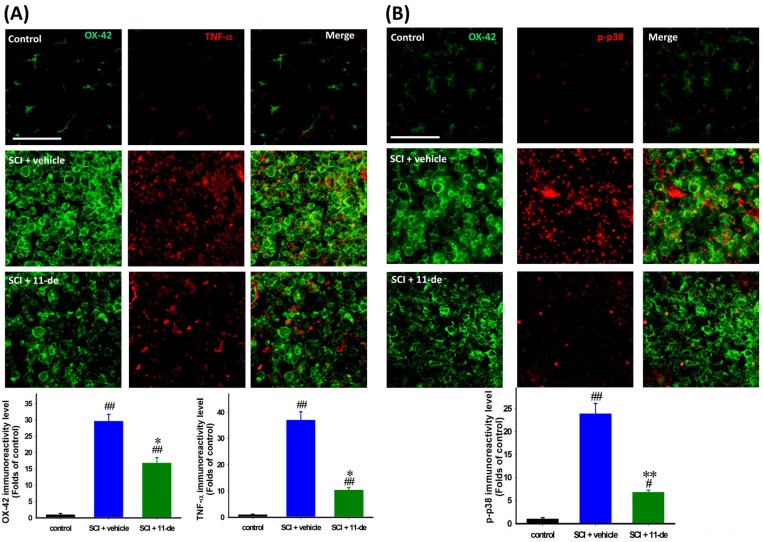
11-Dehydrosinulariolide attenuated SCI-induced inflammation. The rats were sacrificed on day 7 after SCI. The proinflammatory cytokine TNF-α (**A**) and inflammation-related MAPK p-p38 (**B**) were examined through immunohistochemistry. 11-Dehydrosinulariolide (i.t.) attenuated SCI-induced microglia activation. The IR signals of TNF-α and p-p38 were attenuated after 11-dehydrosinulariolide administration. The results also demonstrated that the anti-inflammatory effect and attenuation of microglia activation may occur through that of p38 phosphorylation (**B**). Data are expressed as the means ± SEMs; # *p* < 0.05 and ## *p* < 0.001 compared with the control group; * *p* < 0.05 and ** *p* < 0.001 compared with the vehicle group. Scale bars = 100 μm.

The rats were sacrificed on day 7 after SCI to examine the anti-inflammatory effect of 11-dehydrosinulariolide. Microglia were identified using the OX-42 antibody and double-stained for tumor necrosis factor-α (TNF-α) and phospho-p38 (p-p38). The morphology of microglia in the control spinal cord was the classic “resting” type, having a compact cell soma with fine, extended, ramified processes. After SCI, the microglia became “activated”, exhibiting a hypertrophic cytoplasm, and the OX-42 IR signal was upregulated. The OX-42 IR signal in the 11-dehydrosinulariolide group was significantly weaker than that in the vehicle group (Figure 5A,B). The TNF-α IR signal in the control spinal cord was weak and difficult to visualize; after SCI, this signal was upregulated, with some of the IR signal being localized at the center of the microglial soma (Figure 5A). 11-Dehydrosinulariolide attenuated SCI-induced upregulation of TNF-α MAPK-p38 has a critical role in detrimental microglial activation and TNF-α production. Hence, p38 may be involved in the anti-inflammatory effect of 11-dehydrosinulariolide. The p-p38 IR signal in the control spinal cord was extremely weak, and this signal was upregulated significantly after SCI. Most of the p-p38 IR signal was localized in the microglial soma. 11-Dehydrosinulariolide attenuated SCI-induced microglial p-p38 upregulation (Figure 5B).

### 2.6. 11-Dehydrosinulariolide Promoted an Alternative Pathway of Microglia Activation after SCI

**Figure 6:**
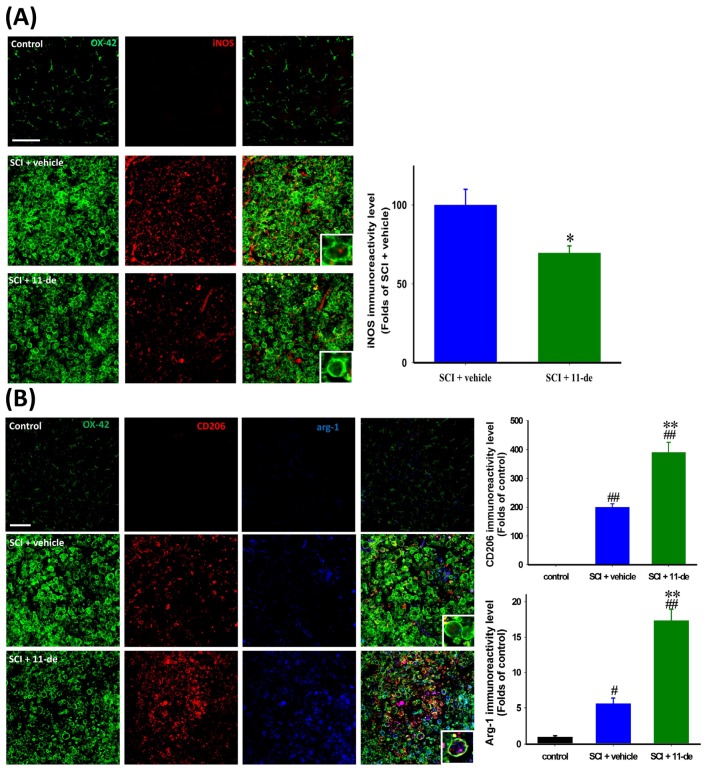
11-Dehydrosinulariolide promoted an alternative pathway of microglia activation after SCI. The rats were sacrificed on day 7 after SCI. The marker of M1 microglia iNOS was detected through immunohistochemistry (**A**). 11-Dehydrosinulariolide administration attenuated SCI-induced iNOS upregulation. The M2 microglia markers CD206 and ARG1 were also detected through immunohistochemistry (**B**). Microglia in both vehicle- and 11-dehydrosinulariolide-treated spinal cords were activated and round. However, 11-dehydrosinulariolide administration enhanced SCI-induced the upregulation of CD206 and ARG1 IR signals. Data are expressed as means ± SEMs; # *p* < 0.05 and ## *p* < 0.001 compared with the control group; * *p* < 0.05 and ** *p* < 0.001 compared with the vehicle group. Scale bars = 100 μm.

To further investigate the possible mechanism of the anti-inflammatory effect of 11-dehydrosinulariolide after SCI, we evaluated M1 and M2 microglial markers on day 7 after SCI. An M1 microglial marker, iNOS, and 2 M2 microglia markers, CD206 and arginase-1 (ARG1), were analyzed. The iNOS IR signal in the control spinal cord was extremely weak compared with that in the injured spinal cord. Thus, we considered the IR signal in the injured spinal cord rather than that in the control spinal cord as 100%. After SCI, the iNOS IR signal was majorly localized in the microglial soma, and 11-dehydrosinulariolide attenuated the iNOS IR signal significantly (Figure 6A). The CD206 IR signal in the control spinal cord was weak and difficult to visualize; after SCI, the CD206 IR signal was upregulated and the cells were round (Figure 6B). The 11-dehydrosinulariolide group showed a significantly enhanced CD206 IR signal compared with the vehicle group. After SCI, the ARG1 IR signal was localized in the soma of OX-42- or CD206-positive microglia, and the 11-dehydrosinulariolide group had significantly enhanced ARG1 IR signals compared with the vehicle group.

## 3. Discussion

In the present study, i.t. pretreatment with a marine-derived compound from a coral culture called 11-dehydrosinulariolide was shown to improve functional recovery after SCI. At 8 h after SCI, 11-dehydrosinulariolide attenuated SCI-induced downregulation of the antiapoptotic protein Bcl-2 and upregulated the cell survival-related pathway proteins p-Akt and p-ERK. Furthermore, it upregulated the transcription factor p-CREB, which regulates Bcl-2 expression, after SCI. On day 7 after SCI, 11-dehydrosinulariolide exhibited anti-inflammatory effects, attenuating SCI-induced upregulation of the proinflammatory proteins iNOS and TNF-α. We also observed that 11-dehydrosinulariolide increased the expression of the M2 microglial markers ARG1 and CD206 in the injured spinal cord on day 7 after SCI.

Because SCI is a sudden and unpredictable traumatic injury, the current strategies focus on secondary injury-specific neuroprotective and anti-inflammatory effects. In acute SCI, both necrosis and apoptotic cell death occur [30,31]. Apoptotic cell death occurs at the lesion after SCI, and neuronal apoptosis is a part of secondary events following SCI. [25,32,33,34]. Enhanced expression of Bcl-2 has a neuroprotective effect after SCI [24,35,36,37]. This evidence suggests that modulating apoptosis may be useful in SCI therapy. Our results revealed that i.t. administration of 11-dehydrosinulariolide enhanced Bcl-2 expression 8 h after injury and, therefore, reduced the number of apoptotic cells (Figure 3).

The PI3K/Akt and ERK pathways are crucial for neuronal cell survival [38,39,40,41,42,43]. Studies have reported p-Akt upregulation at neurons near the lesion site after stroke [44,45,46] and SCI [24,47]. ERK activation has also been observed after stroke [48,49,50], spinal cord ischemia [51], and SCI [24,52,53]. These above-mentioned studies suggested that the PI3K/Akt and ERK pathway activation is an endogenous neuroprotective response after CNS injury. However, this endogenous protection did not last for a long period. The protection was short lasting, Akt was activated at 8 h, but this activation decreased from 24 to 48 h [24,54]. ERK declined from 4 h following SCI [24,52]. Therefore, the enhancement of Akt [24,55,56,57,58,59] and ERK [52,53,60,61,62] activation by pharmaceutical intervention revealed neuroprotective effects after CNS injury. The p-Akt IR signal, not being colocalized with the NeuN IR signal, may be related to glial scar formation [63]. Akt [64] and ERK [62] activate the transcription factor CREB through phosphorylation, which then upregulates Bcl-2 expression [26,27,65,66]. Studies have also suggested that enhanced Akt and ERK activation improves recovery after SCI [24,54]. Therefore, Akt and ERK activation may activate CREB and enhance Bcl-2 expression, thus attenuating apoptosis. 11-Dehydrosinulariolide enhanced the phosphorylation of Akt, ERK, and CREB at 8 h after injury. We previously reported that the in vitro neuroprotective effect of 11-dehydrosinulariolide is mediated through PI3K regulation [23]. This evidence suggested that 11-dehydrosinulariolide offsets SCI-induced apoptosis by enhancing Bcl-2 expression through Akt- and ERK-dependent CREB activation (Figure 3 and Figure 4).

Microglia release NO [67], IL-1β and TNF-α [68], all of which contribute to neuronal dysfunction and cell death in response to injury [69,70,71]. These inflammatory factors are rapidly upregulated and contribute to microglial activation after SCI [72,73,74]. TNF-α initiates several downstream signal transduction pathways such as the NF-κB and MAPK pathways [75]. Increased expression of iNOS is also regulated through the MAPK pathway by p38 and JNK [67,76,77]. Thus, microglial activation and microglia-related of proinflammatory proteins (TNF-α and iNOS) are involved in neuroinflammation after SCI. The therapeutic effect of 11-dehydrosinulariolide observed in the present study may be associated with attenuated SCI-induced activation of microglia (Figure 6).

Proinflammatory cytokines (e.g., TNF-α) are upregulated by microglia during the initial few days after SCI [78]. TNF-α facilitates the development and persistence of secondary injury after SCI [79,80] and induces oligodendrocyte apoptosis [80,81,82], which leads to massive demyelination [83,84]. Therefore, the reduction in spinal cord tissue loss and demyelination after 11-dehydrosinulariolide administration may be due to the reduction of TNF-α levels (Figure 5 and Figure 6B). The members of the MAPK family ERK, JNK, and p38 are responsible for the production of TNF-α [85,86]. In particular, p38 has a critical role in detrimental microglial activation and iNOS and TNF-α production [87,88]. Here, 11-dehydrosinulariolide administration attenuated microglial p38 activation after SCI. Thus, the anti-inflammatory effect of 11-dehydrosinulariolide may be associated with p38-related pathways (Figure 6).

To further investigate the possible anti-inflammatory mechanisms of 11-dehydrosinulariolide after SCI, we evaluated M1 and M2 microglial markers on day 7 after SCI. M1 microglial markers include iNOS, COX-2, and cell surface markers such as CD16 and CD86, whereas those of M2 microglia include CD206, ARG1, transglutaminase-2, and YM1 [16,89]. In the presence of arginine, a common substrate of ARG1 and iNOS, ARG1 downregulates NO production [90]. Therefore, ARG1 and iNOS represent common markers for the M1 and M2 phenotypes [91]. Studies have shown that M1 microglia dominate the microglia population approximately one week after injury to the CNS [92,93] or SCI [12]. We also observed that M1 microglia dominated the microglia population in the vehicle group on day 7 after SCI (Figure 6A). M1 microglia may contribute to the secondary tissue damage following SCI [12,94], whereas M2 polarization may underlie the protective effect. Enhancing M2 microglia polarization in the spinal cord after injury provides neuroprotection. For example, minocycline administration restored impaired neurogenesis by selectively ablating the function of M1-polarized microglia [14,95]. In the present study, the quantification of CD206 and ARG1 IR signals on day 7 after SCI indicated that 11-dehydrosinulariolide increased the number of M2-polarized microglia and reduced that of M1-polarized microglia relatively. Cytokines are one of the factors affecting microglia polarization. The classically activated microglia form after exposure to interferon-γ and TNF-α, leading to M1 microglia formation. In contrast, alternatively “activated” microglia form after exposure to IL-4, IL-10, IL-13, and TGF-β, leading to M2 microglia formation [16,18,89]. In a future study, we will examine whether the M2 polarization-promoting effect of 11-dehydrosinulariolide on microglia is directly affected by the microenvironment conditions. Cyclic AMP and CREB are key regulators of M1-to-M2 microglial transition [96,97,98]. Because 11-dehydrosinulariolide enhanced CREB activation in injured spinal neurons in the present study. We will also examine whether the M2 polarization-promoting effect of 11-dehydrosinulariolide on microglia is affected by the CREB activation in microglia in a future study.

Compared with terrestrial natural products, marine natural products are more chemically novel [99]; approximately 71% of the molecular scaffolds in the Dictionary of Marine Natural Products are present exclusively in marine organisms [100]. This evidence suggests that marine natural products have the potential for further development into drugs. Since 2004, after the established marine-derived drug ziconotide (ω-conotoxin MVIIA) was approved by the US FDA, several marine-derived drugs have been developed; among which 8 are currently available in the market, and 9 are under development [101]. Because the production of marine natural products majorly depends on harvest from wild sources, sustainability and replicability are the bottlenecks in marine-derived drug development [100,102]. These bottlenecks may be resolved through aquaculture, where marine organisms are continuously cultured under well-controlled environmental conditions. *S. flexibilis* is aquacultured in the National Museum of Marine Biology and Aquarium, and the 11-dehydrosinulariolide yield is 100 mg/kg coral (dry weight). The quality and quantity of this compound is sufficient to support future drug development.

Pretreatment of SCI rats with 11-dehydrosinulariolide has led to antiapoptotic and anti-inflammatory effects. The antiapoptotic effect may be due to PI3K/Akt-dependent CREB activation, whereas the anti-inflammatory effect may be due to M2 microglia polarization. However, the detailed mechanism regarding the regulation of M2 microglia polarization by 11-dehydrosinulariolide requires further investigation. We will perform further research on 11-dehydrosinulariolide, including mechanism identification, chemical modification, and formulation, to enhance its therapeutic effect. The future study is supported by Taiwan’s National Research Program for Biopharmaceuticals and is in progress.

## 4. Materials and Methods

### 4.1. Implantation of i.t. Catheters and Spinal Cord Contusion Injury

Female Wistar rats (weight, 300–320 g) were used in the experiments. The surgical procedures were performed as previous described [63,103]. Briefly, i.t. catheters were inserted via the atlanto-occipital membrane into the i.t. space at the level of T12. After a 4-day recovery period after implantation of the i.t. catheter, the spinal cord contusion injury surgery was performed. Rats were subjected to spinal cord contusion injury at T10 using a New York University (NYU) impactor (W.M. Keck Center for Collaborative Neuroscience, Rutgers University, New Brunswick, NJ, USA). Bladder evacuation was applied twice daily for at least 7 days. The use of animals conformed to the Guiding Principles for the Care and Use of Animals published by the American Physiological Society and was approved by the National Sun Yat-Sen University Animal Care and Use Committee (Kaohsiung, Taiwan). All efforts were made to minimize the number of animals used and their suffering.

### 4.2. Drug Treatment

After implantation of i.t. catheters, the rats were randomly divided into 3 groups, as follows: Control group: the rats received i.t. injection of normal saline (10 μL). SCI + vehicle group: rats pretreatment normal saline for 2 days and once a day after SCI for 5 days (10 μL). SCI + 11-dehydrosinulariolide group: rats pretreatment 11-dehydrosinulariolide (0.1, 1, or 5 μg/10 μL) for 2 days and once a day after SCI for 5 days.

### 4.3. Behavioral Analysis

The Basso-Beattie-Bresnahan (BBB) locomotion scale [104] was used to assess the improvement in hindlimb function in the treated animals with SCI (*n* = 5 per group). The BBB scores were documented by independent examiners who were unaware of the experimental groups.

### 4.4. Immunohistochemistry and Image Analysis

The immunohistochemical procedures and the quantification of immunochemical images were performed as previous described [63,103,105,106,107]. Briefly, under completely anesthetized by isoflurane, the rats (*n* = 4 per each group per time point) were intracardially perfused with cold phosphate-buffered saline (PBS) (pH 7.4) with heparin (0.2 U/mL) and followed by 4% paraformaldehyde in PBS. The T8–T12 thoracic vertebrae were collected. The spinal cords collected at the different time points from different treatment groups were then mounted on the same tissue block in Tissue-Tek O.C.T. (optimal cutting temperature) compound (Sakura Finetek Inc., Torrance, CA, USA) to decrease the variation across following procedures. Next, 20-μm-thick sections were cut on a cryostat (HM550; Microm, Waldorf, Germany) at −30 °C, air-dried at room temperature for 1 h, and incubated with 4% normal horse serum diluted in 0.01% Triton X-100 and PBS (pH 7.4) for 1 h. The sections were incubated with primary antibodies (Table 1) overnight at 4 °C.

The sections were then incubated with secondary antibodies (Cy™ 3-labeled donkey anti-rabbit antibody; Jackson ImmunoResearch Laboratories Inc., West Grove, PA, USA and/or Alexa Fluor 488-labeled chicken anti-mouse antibody; Molecular Probes Inc., Eugene, OR, USA) at room temperature for 1 h. The images were inspected using a Leica TCS SP5II fluorescence microscope (Leica, Wetzlar, Germany) and captured using a SPOT Xplorer Digital camera (Diagnostic Instruments Inc., Sterling Heights, MI, USA). The exposure times were the same for all spinal cord sections mounted on the same microscope slide.

For the SCI group, sections located at approximately 400 μm rostral to the lesion epicenter (with the largest cavity size as) was selected from a series of consecutive sections, and three sequential sections were measured. Four square regions of interest (ROIs, 200 μm × 200 μm) were placed on each section near the lesion border. For uninjured spinal cords, immunofluorescence was acquired from whole portions of the spinal cord sections. The MetaVue Imaging software (Molecular Devices Corporation, Downingtown, PA, USA) was used to calculate the pixel values of the positive area (density value/unit area). Immunohistochemical data were shown as fold change relative to the control group or the SCI + vehicle group, which was considered to represent a fold change of 1.

### 4.5. TUNEL Staining

The TUNEL assay was performed by a commercially available kit (In Situ Cell Death Detection Kit; Roche Diagnostics, Mannheim, Germany). Sections were prepared in the same manner as the sections used for immunohistochemistry. The sections were incubated in permeabilisation solution (0.1% Triton X-100 in 0.01% sodium citrate) for 2 min on ice. Then the sections were incubated with TUNEL reaction mixture solution for 60 min at 37 °C. The sections were analyzed under Leica TCS SP5II fluorescence microscope (Leica) and captured using a SPOT Xplorer Digital camera (Diagnostic Instruments Inc.).

### 4.6. Eriochrome Cyanine Myelin Staining

Spinal cord cryosections were prepared in the same manner as the sections used for immunohistochemistry. After being air-dried at room temperature and fixed by 4% paraformaldehyde in PBS for 10 min, the sections were incubated with 0.16% eriochrome cyanine-R (Sigma-Aldrich, St. Louis, MO, USA) and 10% FeCl_3_·6H_2_O (Sigma-Aldrich) in 0.5% H_2_SO_4_ (Sigma-Aldrich) for 30 min at room temperature. The sections were then washed with running tap water and differentiated in 1% ammonium hydroxide followed by counterstained with neutral red (Sigma-Aldrich). The areas of spared white matter were calculated by subtracting the cavity areas and gray matter from the total spinal cord sectional areas using MetaVue Imaging software (Molecular Devices Corporation). The portion of spared white matter was calculated as follows: (spared white matter/total area of spinal cord) × 100.

### 4.7. Western Blotting

The western blotting procedures and the quantification were performed as previous described [63,106,107,108]. Briefly, under complete anesthesia with isoflurane, the rats (*n* = 4/group) were intracardially perfused with cold PBS (pH 7.4) with heparin (0.2 U/mL), protease inhibitor (Complete ULTRA; Roche Diagnostics), and phosphatase inhibitor (PhosSTOP; Roche Diagnostics). Then a 1-cm-long spinal cord covering the injury site was homogenized in lysis buffer on ice. Protein concentrations were determined using the DC protein assay kit (Bio-Rad, Hercules, CA, USA). An equal volume of sample buffer (2% sodium dodecyl sulphate (SDS), 10% glycerol, 0.1% bromophenol blue, 2% 2-mercaptoethanol and 50 mM Tris-HCl, pH 7.2) was added to the sample, which was then loaded onto a tricine SDS-polyacrylamide gel and electrophoresed at 150 V for 90 min. The proteins were transferred to a polyvinylidene difluoride membrane (PVDF membrane; Immobilon-P, EMD Millipore, Bedford, MA, USA). After blocking for 1 h with 5% nonfat milk in Tris-buffered saline at room temperature, the PVDF membrane was subsequently incubated overnight with primary antibodies (Table 1) at 4 °C.

The immunoreactive bands were visualized by ECL kit (Millipore) and by the UVP Imaging System (BioChemi; UVP, Upland, CA, USA). The relative densitometric quantification was performed using LabWorks 4.0 software (UVP). The relative variations of the protein of interest between the bands of the various treatment groups and of the control group were calculated from the same blot image.

### 4.8. Statistical Analysis

All data are presented as the mean ± the standard error of the mean (SEM). Behavioral outcomes after SCI were analyzed by two-way repeated-measures analyses of variance (ANOVA) with treatment and time point as independent variables followed by post-hoc pairwise multiple comparisons using the Student-Newman-Keuls method. For western blotting and immunohistochemical analyses, significant differences between the treatment groups were calculated using one-way ANOVA followed by Student-Newman-Keuls post hoc tests for multiple comparisons. A *p* value of < 0.05 was considered statistically significant.

## 5. Conclusions

Present findings demonstrate that 11-dehydrosinulariolide has antiapoptotic and anti-inflammatory properties in SCI-rats. The antiapoptotic effect may be due to PI3K/AKT cell survival pathway activation, whereas the anti-inflammatory effect may be due to alternation of microglia activation. We concluded that 11-dehydrosinulariolide ameliorated SCI-induced motor dysfunction. Hence, the soft coral-derived compound 11-dehydrosinulariolide to be identified as a potential candidate for future development as a therapeutic agent for the treatment of neuronal injury.

## Figures and Tables

**Table 1:** The antibodies used in this study.

Antibody	Supplier	Catalog No.	Host	Application
ARG1	Santa Cruz Biotechnology (Dallas, TX, USA)	Sc-18351	goat	IHC
β-actin	Sigma (St. Louis, MO, USA)	A5441	mouse	western
Bcl-2	BD Biosciences (San Jose, CA, USA)	610539	mouse	IHC, western
p-Akt	Cell Signaling Technology (Danvers, MA, USA)	4060	rabbit	IHC, western
Akt	Cell Signaling Technology (Danvers, MA, USA)	9272	rabbit	western
Cd11b (OX-42)	Serotec (Raleigh, NC, USA)	MCA275	mouse	IHC
Cd206	Abcam (Cambridge, MA, USA)	Ab64693	rabbit	IHC
p-CREB	Cell Signaling Technology (Danvers, MA, USA)	9198	rabbit	IHC, western
CREB	Cell Signaling Technology (Danvers, MA, USA)	9197	rabbit	western
p-ERK	Cell Signaling Technology (Danvers, MA, USA)	9101	rabbit	IHC, western
ERK	Cell Signaling Technology (Danvers, MA, USA)	9102	rabbit	western
iNOS	EMD Millipore (Bedford, MA, USA)	Ab5382	rabbit	IHC
NeuN	EMD Millipore (Bedford, MA, USA)	MAB377	mouse	IHC
NeuN	EMD Millipore (Bedford, MA, USA)	ABN78	rabbit	IHC
p-p38	Cell Signaling Technology (Danvers, MA, USA)	4511	rabbit	IHC

## Supplementary Materials

The following are available online at www.mdpi.com/1660-3397/14/9/160/s1, Figure S1: Co-treatment with 11-dehydrosinulariolide improved locomotor recovery in rats with SCI. The rats were treated with 11-dehydrosinulariolide immediately after SCI and on the following 6 days (once daily; total, 7 times; *n* = 2). Their behavior showed that the co-treatment process enhanced the recovery after SCI (A). However, the AUC of the co-treatment group was similar to that of the 1-μg group (180 and 193, respectively) * *p* < 0.05 compared with the vehicle group.
