# Supplementary material for: A Coral-Derived Compound Improves Functional Recovery after Spinal Cord Injury through Its Antiapoptotic and Anti-Inflammatory Effects
Source: Mar Drugs. 2016 Sep 2;14(9):160. doi: 10.3390/md14090160 (PMC5039531; doi:10.3390/md14090160)
Supplement: Supplementary file 1 [file marinedrugs-14-00160-s001.pdf]

Supplementary Materials: A Coral-Derived Compound Improves Functional Recovery after Spinal Cord Injury through Its Antiapoptotic and Anti-Inflammatory Effects

Chun-Hong Chen, Nan-Fu Chen, Chien-Wei Feng, Shu-Yu Cheng, Han-Chun Hung, Kuan-Hao Tsui, Chi-Hsin Hsu, Ping-Jyun Sung, Wu-Fu Chen and Zhi-Hong Wen

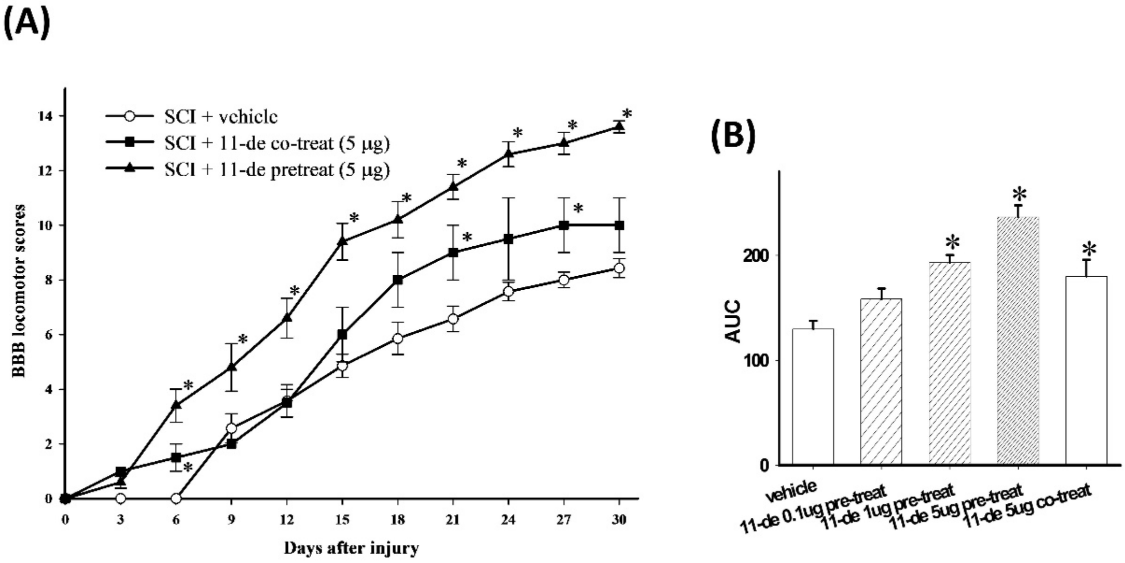

Figure S1. Co-treatment with 11-dehydrosinulariolide improved locomotor recovery in rats with SCI. The rats were treated with 11-dehydrosinulariolide immediately after SCI and on the following 6 days (once daily; total, 7 times; $n = 2$). Their behavior showed that the co-treatment process enhanced the recovery after SCI (A). However, the AUC of the co-treatment group was similar to that of the 1-μg group (180 and 193, respectively) * $p < 0.05$ compared with the vehicle group.
